# Effect of Non-nucleoside Resistance-Associated Mutations on the Effectiveness of Long-Acting Cabotegravir + Rilpivirine Therapy: Insights From the Real-World RELATIVITY Cohort

**DOI:** 10.1093/ofid/ofag426

**Published:** 2026-07-07

**Authors:** David Rial-Crestelo, Jara Llenas-García, Carmen Hidalgo-Tenorio, Jesús Troya García, María José Galindo Puerto, María Remedios Alemán Valls, Miguel Torralba González de Suso, Luis Buzón, Alberto Díaz de Santiago, Adrián Rodríguez, Sonia Calzado Isbert, María Aguilera García, Luis Enrique Morano Amado, David Vinuesa García, Enrique Bernal Morell, Rosa María Martínez Álvarez, Noemí Cabello-Clotet, Analuz Fernández, María del Carmen Montero Hernández, Cristina Díez Romero, Ruth Calderón Hernaiz, Desiree Pérez Martínez, Laura Gisbert Pérez, Víctor Arenas García, Cristina Escrich, María Carmen Fariñas Álvarez, Ana Lucas Dato, Juan Emilio Losa García, Hadrián Pernas Pardavila, Juan Carlos Gainzarain, Miriam Estébanez, Patricia Barragán Gallo, Marta Clavero Olmos, Miguel Vicente Egido Murciano, Oscar Luis Ferrero Benéitez, Roberto Pedrero-Tomé, Alfonso Cabello-Úbeda, Luz Martín-Carbonero, RELATIVITY studygroup, Ana Cristina Antolí Royo, Carmen Grande Sáez, María Ángeles Garcinuño Jiménez, Ana Carrero Gras, Eva María Ferreira Pasos, Pablo Bachiller Luque, Hadrián Pernas Pardavila, Mercedes González, Nuria Vazquez Temprano, Miriam Estébanez, Ana Muñoz Gómez, Jose Reynaldo Homen Fernandez, Juncal Pérez-Somarriba Moreno, Maravillas Carralón, María José Núñez Orantos, Maria Nieves Sanz Perez, Noemí Cabello Clotet, Susana Olmedo Hernández, Valeria Cabral Sousa, Vicente Estrada, Virginia Víctor Palomares, Ana Ferrer Ribera, Andreu Belmonte, Carolina Pinto, María José Galindo Puerto, Rosa Oltra Sempere, Sandra Pérez Gómez, Álvaro Cecilio, Isabel Sanjoaquin Conde, Maria Jose Crusells Canales, Pilar Jimenez Marcen, Ana Lucas Dato, Belén Martínez López, Inmaculada González Cuello, Jara Llenas-García, María García López, Carolina Navarro, Luis Buzón, María Fernandes, Amparo Exojo Morales, Carlos de Andrés David, Inmaculada Poquet Catalá, Karenina Antelo Cuéllar, Laia Navarro Peiró, María Josefa de la Asunción Villaverde, Silvia Lope Bolumar, Alba León Barbosa, Esther Nogales Nieves, Eva García Alcalde, Juan José Corte García, Lucía Alonso Alonso, María Vanessa López Peláez, Sara Jaber Carballo, Albert Gómez Lozano, Sara García Torras, Marta Milián Sanz, Ana Isabel Lérida Urteaga, Antonia Pérez Barja, Javier López-Nieto Sempere, Magdalena Muelas Fernández, Beatriz de la Calle, Ángela Botella Zaragoza, Félix Gutiérrez Rodero, Javier García Abellán, Mar Masiá, Paula Mascarell Arlandis, Sergio Padilla Urrea, Chiara Fanciulli, Cristina Diez Romero, Francisco Tejerina Picado, Juan Carlos López Bernaldo de Quiros, Leire Pérez Latorre, Mª Teresa Aldamiz-Echevarría Lois, Antonio Jesús Sánchez Guirao, Ángeles Muñoz Perez, Antonia Alcaraz García, Cristina Tomás Jimenez, Elena Guijarro Westermeyer, Enrique Bernal Morell, Eva García Villalba, Joaquín Bravo Urbieta, María Dolores Hernández Lorente, Rodrigo Martínez Rodríguez, Román Gonzalez Hipólito, Noemí Ramos Vicente, Patricia Barragán Gallo, Alberto Juárez Toquero, Beatriz Valentín Casado, Jesica Abadía Otero, Julia Gómez Barquero, Pablo Bachiller Luque, Yolanda Arranz García, Adriana Pinto, David Rial, Federico Pulido, Juan Martín, Laura Bermejo Plaza, María De Lagarde, María Teresa López Caballero, Mireia Santacreu, Otilia Bisbal, Rafael Rubio, Roser Navarro, Victoria Macheño del Real, Celia Miralles Álvarez, Guillermo Pousada, Henar de las Heras Miralles, Luis Enrique Morano Amado, Víctor Asensi, Laura Guio Carrión, Mikel del Álamo, Ester Sáez de Adana Arroniz, Juan Carlos Gainzarain Arana, Ana Vegas Serrano, Juan Emilio Losa García, María Velasco Arribas, Maite Ganchegui Aguirre, Miriam López Martínez, Óscar Luis Ferrero Beneitez, Alicia Sedo Mor, Juan Tiraboschi, Josefa Soler González, Víctor Arenas García, Nereyda Tosco García, Remedios Alemán Valls, Guillermo Soria Fernández-Llamazares, Juan Víctor San Martín López, Ruth Calderón Hernaiz, Alberto Delgado Fernández, Lorenzo Sánchez, Miguel Torralba, Alberto Romero Palacios, Maria Antonia Sepúlveda, Alejandra Gimeno García, Alejandro David Bendala Estrada, Carmen Montero Hernández, Diana Corps Fernández, Jose Carlos Escribano Stablè, María Del Mar Garcia Navarro, Marouane Menchi Elanzi, Ana Cerezales Calviño, Bárbara Alonso Moreno, José Fernando Lluch Perales, Alfonso Cabello, Miguel Górgolas Hernández-Mora, María Rubio Olivera, Marta Clavero Olmos, Belén Escribano Losada, Guillermo Cuevas, Jesús Troya García, Mariano Matarranz, Pablo Ryan, Roberto Pedrero Tomé, Ana Belén Delgado Hierro, Carmen Bucsa, Jose Ignacio Bernardino, Juan González García, Luis Ramos Ruperto, Luz Martín Carbonero, María Del Mar Arcos Rueda, María Eulalia Valencia, María Luisa Montes, Rafael Micán, Rocío Montejano, Andoni Casen Gil, Ignacio de los Santos, María Aguilera García, Aitziber Illaro Uranga, Carlos Armiñanzas Castillo, Claudia González Rico, Francisco Arnaiz de las Revillas Almajano, Manuel Gutiérrez Cuadra, Maria Carmen Fariñas Álvarez, Noelia Ruiz Alonso, Ángela Forcén Vicente de Vera, María Aranzazu Caudevilla Martínez, Rosa María Martínez Álvarez, Ruth Caballero Asensio, David Dalmau Juanola, Laura Gisbert Pérez, Mireia Cairó Llobell, Roser Font Canals, Xavier Martínez Lacsa, Jose Sanz, Alberto Díaz de Santiago, Ana Fernández Cruz, Sara de la Fuente Moral, Ana Moreno, Daniel de las Heras Gómez, María Jesús Pérez Elías, María Jesús Vivancos Gallego, Mario Pons Guillén, Desirée Pérez Martínez, Maria Elisa Pino Diaz, Miguel Alberto de Zárraga Fernández, Miguel Vicente Egido Murciano, Teresa Omiste Sanvicente, Adrián Ferré, Antoni Campins, Francisco Fanjul, Luisa Martín Pena, Melchor Riera, Adrián Rodríguez, Patricia Sorní Moreno, Carmen Hidalgo, Sergio Sequera Arquelladas, Cristina Escrich, María Del Carmen Navarro

**Affiliations:** Hospital Universitario 12 de Octubre, imas12 Health Research Institute, Madrid, Spain; CIBERINFEC, Instituto de Salud Carlos III, Madrid, Spain; CIBERINFEC, Instituto de Salud Carlos III, Madrid, Spain; Hospital Universitario La Paz, IdiPAZ Health Research Institute, Madrid, Spain; Miguel Hernández University, Elche, Spain; Hospital Universitario Virgen de las Nieves, Instituto de Investigación Biosanitaria de Granada (IBS-Granada), Granada, Spain; Hospital Universitario Infanta Leonor, Madrid, Spain; Hospital Clínico Universitario de Valencia, Valencia, Spain; Hospital Universitario de Canarias, Tenerife, Spain; IDISCAM, Hospital Universitario de Guadalajara, Guadalajara, Spain; Universidad de Alcalá, Alcalá de Henares, Madrid, Spain; Hospital de Burgos, Burgos, Spain; Hospital Universitario Puerta de Hierro, Madrid, Spain; Hospital Universitario Son Llàtzer, Health Research Institute of the Balearic Islands (IdISBa), Palma de Mallorca, Spain; Parc Taulí Hospital Universitari, Sabadell, Spain; Institut D’Investigació I Innovació Parc Taulí (I3PT), Sabadell, Spain; Universitat Autònoma de Barcelona, Barcelona, Spain; Hospital Universitario de La Princesa, Madrid, Spain; Álvaro Cunqueiro University Hospital, Vigo, Spain; Hospital Clínico San Cecilio-PTS, Granada, Spain; Hospital General Universitario Reina Sofía, Murcia, Spain; Hospital Universitario Miguel Servet, Zaragoza, Spain; CIBERINFEC, Instituto de Salud Carlos III, Madrid, Spain; Hospital Clínico San Carlos, Madrid, Spain; Instituto de Investigación Sanitaria San Carlos (IdISSC), Madrid, Spain; Universidad Complutense de Madrid, Madrid, Spain; Bellvitge University Hospital, Barcelona, Spain; Bellvitge Biomedical Research Institute (IDIBELL), Barcelona, Spain; University of Barcelona, Barcelona, Spain; Hospital Universitario de Torrejón, Madrid, Spain; Universidad Francisco de Vitoria, Madrid, Spain; CIBERINFEC, Instituto de Salud Carlos III, Madrid, Spain; Hospital General Universitario Gregorio Marañón, Madrid, Spain; Instituto de Investigación Sanitaria Gregorio Marañón (IiSGM), Madrid, Spain; Hospital Universitario de Fuenlabrada, Madrid, Spain; Hospital Universitario San Agustín, Avilés, Spain; Hospital Universitario Mutua Terrassa, Barcelona, Spain; Hospital Universitario de Cabueñes, Gijón, Spain; Hospital Verge de la Cinta, Tortosa, Spain; Hospital Universitario Marqués de Valdecilla, Santander, Spain; Hospital Comarcal de la Vega Baja, Alicante, Spain; Hospital Universitario Fundación Alcorcón, Madrid, Spain; Universidad Rey Juan Carlos, Madrid, Spain; Complexo Hospitalario Universitario de Pontevedra, Instituto de Investigación Sanitaria Galicia Sur, Pontevedra, Spain; Hospital Universitario de Álava, Vitoria-Gasteiz, Spain; Hospital Central de la Defensa Gómez Ulla, Madrid, Spain; Hospital Residencia Sant Camil, Barcelona, Spain; Hospital Universitario Infanta Elena, Madrid, Spain; Hospital Universitario San Jorge, Huesca, Spain; Hospital Universitario de Basurto, Bilbao, Spain; Fundación Para la Investigación e Innovación Biomédica (FIIB) de los Hospitales Universitarios Infanta Leonor (Vallecas) y Sureste (Arganda del Rey), Madrid, Spain; Division of Infectious Diseases, Hospital Universitario Fundación Jiménez Díaz, IIS-FJD, Universidad Autónoma de Madrid, Madrid, Spain; CIBERINFEC, Instituto de Salud Carlos III, Madrid, Spain; Hospital Universitario La Paz, IdiPAZ Health Research Institute, Madrid, Spain

**Keywords:** long-Acting, NNRTI resistance-associated mutations, real-world, rilpivirine

## Abstract

**Background:**

The impact of pre-existing non-nucleoside reverse transcriptase inhibitor (NNRTI) resistance-associated mutations (RAMs) on the effectiveness of long-acting (LA) cabotegravir (CAB) and rilpivirine (RPV) remains poorly defined, particularly for mutations like K103N that do not directly compromise RPV susceptibility (non-RPV NNRTI RAMs).

**Methods:**

This observational substudy within the Spanish RELATIVITY cohort included people with HIV (PWH) who switched to CAB + RPV LA before 2025. We compared virologic outcomes between those with non-RPV NNRTI RAMs and a reference group without NNRTI/INSTI-RAMs or prior NNRTI-based virologic failure (VF). An additional subgroup with RPV-associated RAMs was also analyzed. Confirmed virologic failure (CVF) was defined as 2 consecutive viral loads ≥ 200 copies/mL or 1 ≥500 copies/mL.

**Results:**

Among 1358 participants, 83 (6.1%) harbored non-RPV NNRTI RAMs (47 with K103N) and 1247 served as the reference group. After a median follow-up of 16.7 months, CVF rates were similar between the non-RPV NNRTI RAMs group and the reference group (1.2% vs 0.8%; HR: 1.39; 95% CI: 0.18–10.84; *P* = .755). No significant differences were observed in viral blips (6% vs 8.2%; *P* = .481). Notably, in the subgroup of 28 participants with RPV-associated RAMs (including 5 with high-level resistance), no CVFs occurred over a median of 13.5 months.

**Conclusions:**

In a real-world setting, CAB + RPV LA demonstrated high effectiveness in PWH with pre-existing NNRTI RAMs that do not compromise RPV susceptibility, including K103N. While no failures were seen in those with RPV-associated RAMs, these results should be interpreted with caution due to the small sample size.

Long-acting (LA) antiretroviral therapy (ART) with cabotegravir (CAB) and rilpivirine (RPV) (CAB + RPV LA) represents a major advance in the management of people with HIV (PWH), offering an alternative to daily oral therapy and potentially improving adherence, treatment satisfaction, and long-term virologic outcomes. Pooled data from clinical trials have demonstrated high levels of efficacy and tolerability of this therapy, with very few reported virologic failures (VF) [1–7]. These findings have already been confirmed in real-world settings by large cohorts, including ours, supporting the virologic effectiveness observed in randomized clinical trials [8–12].

Several studies have examined the influence of pre-existing antiretroviral resistance on the effectiveness of CAB + RPV LA [13–17]. In particular, non-nucleoside reverse transcriptase inhibitor (NNRTI) resistance-associated mutations (RAMs) represent a major concern as they may reduce RPV susceptibility, even when they do not directly involve RPV-associated resistance pathways. Although subject harboring the NNRTI RAMs K103N were allowed to be included in the pivotal FLAIR, ATLAS, and ATLAS-2 M studies evaluating LA CAB/RPV, the actual number of participants with K103N mutations and their outcomes have not been reported separately to date [4–6]. Indeed, the European product label for CAB + RPV LA contraindicates its use in the presence of any NNRTI RAMs [18]. Therefore, the clinical relevance of NNRTI RAMs that do not compromise RPV susceptibility (non-RPV NNRTI RAMs) remains poorly defined. This gap in evidence is especially relevant in real-world settings, where historical genotypic resistance tests (GRT), prior antiretroviral exposures, and archived viral variants can be heterogeneous.

Understanding whether these mutations influence treatment outcomes is essential for refining patient selection criteria, optimizing the use of LA-ART, and aiding resistance-based decision-making. The RELATIVITY cohort, a nationwide Spanish study cohort in real-world settings, offers a unique opportunity to evaluate the impact of non-RPV NNRTI RAMs on the virologic performance of CAB + RPV LA regimen.

This study aimed to assess the effectiveness of CAB + RPV LA in individuals harboring NNRTI RAMs by comparing virologic outcomes with those of individuals without such mutations. By characterizing the real-world implications of these resistance patterns, we aim to provide evidence to guide clinical decisions and support the safe expansion of LA treatment strategies.

## METHODS

### Study Design and Setting

This was an ambispective substudy nested within the nationwide, multicenter RELATIVITY cohort encompassing 58 Spanish hospitals in a real-world clinical practice. The study was launched in 2023, and monitoring is expected to continue until 2029. Patients who switched to CAB + RPV LA before January 2025, were included and followed up prospectively. This substudy was designed to evaluate the effectiveness of CAB + RPV LA in specific patient subgroups defined by their historical GRT. Data were collected retrospectively and prospectively during follow-up in accordance with the standardized procedures established in the parent study. The RELATIVITY methodology has been reported elsewhere [19]. Overall, the analysis comprised 1 reference group without NNRTI RAMs and 3 exposure groups defined according to historical NNRTI resistance patterns (non-RPV NNRTI RAMs, K103N mutation, and RPV-associated NNRTI RAMs). Each exposure group was compared separately with the same reference population depending on the outcome analyzed.

### Participants

Eligible participants were adults aged ≥18 years with HIV-1 who initiated CAB + RPV LA and were virologically suppressed at baseline (defined as an VIH-1 viral load <50 copies/mL). For this substudy, the inclusion criteria required the availability of at least 1 historical GRT. The exclusion criteria were infection with HIV-1 subtype A6, historical or documented resistance to second-generation integrase inhibitors (INSTIs), pregnancy, and participation in clinical trials involving CAB + RPV LA.

For the primary analysis, the exposed group comprised participants with a history of NNRTI RAMs that were not predicted to affect RPV susceptibility (non-RPV NNRTI RAMs). For the secondary analysis, we considered 2 additional groups: 1 with only participants with the K103N mutation and another with participants with NNRTI mutations predicted to affect RPV susceptibility (RPV NNRTI RAMs).

The reference group comprised participants who met all the following criteria: (1) availability of a historical GRT; (2) Absence of NNRTI across all available genotypes; and (3) no prior VF while receiving an NNRTI-based regimen, even when historical genotypes showed no NNRTI resistance. These criteria were established to ensure a reference population without known genotypic factors that could compromise the effectiveness of CAB + RPV LA.

### Data Collection

Data for the substudy were obtained through a combination of retrospective data extraction and prospective data collection. Study data were collected and managed using Research Electronic Data Capture (REDCap), hosted at Infanta Leonor University Hospital. The information collected included demographic data, anthropometric measurements, HIV-related information, laboratory data, treatment information (injection schedule, type and site of injection, and oral bridge therapies), and follow-up. There was no established follow-up schedule; each hospital followed its own standard procedures for assessment in accordance with national and international guidelines.

All data collection procedures adhered to the standardized protocols established in the RELATIVITY study to ensure consistency and reliability. For participants with NNRTI mutations, historical and current resistance profiles were meticulously reviewed. All these RAMs must have been detected in RNA while HIV was replicating. Each identified mutation was cross-referenced using the Stanford University HIV Drug Resistance Database (HIVdb) to obtain standardized resistance scores and clinical susceptibility interpretations for all NNRTI agents. Furthermore, participating centers were requested to provide granular longitudinal data for these cases, including whether the genotype was performed at baseline or after VF or treatment interruption. For nonbaseline genotypes, the specific antiretroviral regimen at the time of VF was also recorded. Data were anonymized and entered into a secure database, and quality checks were performed regularly to minimize missing or inconsistent information.

### Variables

Baseline characteristics included age, sex at birth, time since HIV diagnosis, HIV transmission route, ART duration, history and presence of VF, baseline and/or failure-associated GRT, antiretroviral regimen, immunological parameters (CD4+, CD8+, and CD4+/CD8+ ratio), and plasma viral load (VL).

To determine which mutations could compromise the efficacy of NNRTIs, we used the most recent IAS–USA HIV drug resistance mutation list as the reference standard [20]. This resource was used to classify NNRTI RAMs and determine their clinical relevance, enabling discrimination between mutations with a potential impact on RPV susceptibility and those considered clinically nonrelevant according to the current expert consensus. NNRTI RAMs were classified and interpreted using the HIVdb version 9.8 scoring system and were categorized as high, intermediate, low, or potential low-level resistance [21].

Confirmed virologic failure (CVF) was defined as 2 consecutive plasma HIV-1 RNA values ≥200 copies/mL or a single measurement ≥500 copies/mL. This definition of VF was deliberately chosen to be more stringent, even though the current consensus set a threshold of a single HIV-1 RNA measurement >1000 copies/mL to define it [22]. A blip was defined as a temporary, single HIV-1 RNA measurement between 50 and 500 copies/mL that returned to undetectable levels on repeat testing without modification of therapy. Treatment discontinuations were categorized as temporary or permanent, and the underlying reasons—including injection-site reactions, systemic adverse events, CVF, drug–drug interactions, or patient preference—were systematically documented.

### Outcomes

The primary objective was to compare the CVF rate in PWH receiving CAB-RPV LA with or without non-RPV NNRTI RAMS. The secondary objectives were to: (1) compare the number of viral blips in patients with and without non-RPV NNRTI-RAMs; (2) compare the rate of CVF in participants with and without a history of the NNRTI mutation K103N; and (3) compare the rate of CVF in participants with and without RPV NNRTI RAMs.

### Statistical Analysis

All analyses were performed using data available until March 2025. Categorical variables were compared using Pearson's χ^2^ test or Fisher's exact test, as appropriate. Continuous variables were assessed using nonparametric tests (Mann–Whitney *U* test for 2-group comparisons). Discontinuations, adverse events, and reasons for treatment changes were compared between groups using tests of proportions. A two-sided *P* value <.05 was considered statistically significant.

Time-to-event analyses were performed to evaluate virological failure and treatment discontinuation according to the NNRTI mutation status. Kaplan–Meier survival curves were constructed and compared using the log-rank test. Hazard ratios (HRs) with 95% confidence intervals (CIs) were estimated using unadjusted Cox proportional hazards models, including only mutation status as the explanatory variable, to provide a quantitative estimate consistent with the Kaplan–Meier analysis.

Participants were censored at the time of treatment discontinuation, loss to follow-up, or at the last available virologic assessment (March 2025). Analyses were performed using R software version 4.5.1 (R Foundation for Statistical Computing, Vienna, Austria), and figures were generated using R and GraphPad Prism version 8.0 (GraphPad Software, San Diego, CA, USA).

### Ethical Aspects

The study was approved by the Ethics Committee for Research with Medicines of the Burgos and Soria Health Area (reference 23-00144). Written informed consent was obtained from all the participants. All data were managed confidentially and in accordance with the applicable Spanish and European Union data protection legislation.

## RESULTS

### Study Population

Of the 3146 participants in the RELATIVITY study who started treatment before January 2025, 1358 (43.17%) had GRT, met all inclusion/exclusion criteria, and were included in this study. Overall, 111 participants (8.2%) harbored at least 1 NNRTI RAMs: 83 (6.1%) without reduced susceptibility to RPV and 28 (2.1%) with a predicted impact on RPV susceptibility. Baseline demographic and clinical characteristics were generally balanced between patients with non-RPV NNRTI RAMs and those in the reference group (Table 1). In brief, the study population was predominantly male (82%) with a median age of 45 years, a long-standing history of HIV infection, and prolonged exposure to ART (11.5 years, IQR: 7.8–19). However, PWH with non-RPV-NNRTIs RAMs had been exposed to ART for a longer time (11.5 vs 8 years; *P* < .001), had worse immunovirological status at diagnosis (CD4 nadir: 286 vs 350; *P* = .008), had more often previous VF (18.3% vs 2.6%, *P* < .001), and had more often archived the RAM 184 V (19.5% vs 0.6%; *P* < .001), compared with the reference group. Among participants with non-RPV NNRTI RAMs, the most frequently observed mutation was K103N, detected in 47 of 83 individuals, either alone or in combination with other NNRTI RAMs. The E138A mutation was observed in 9 participants. Supplementary Table 1 provides additional baseline characteristics of participants with RPV-associated mutations, respectively.

**Table 1.** Baseline Characteristics According to NNRTI Mutations Not Affecting Rilpivirine Susceptibility

	Non-RPV NNRTI RAMs (n = 83)	Reference Group (n = 1 247)	*P* Value
Age (y), median (IQR)	45 (37.8–53.4)	44 (37–52)	.335
BMI (kg/m^2^), median (IQR)	24.5 (22.5–26.7)	25 (22.4–27.8)	.329
BMI ≥ 30 kg/m^2^, *n* (%)	8 (10.5)	167 (14.6)	.323
Oral lead-in therapy, *n* (%)	7 (8.4)	103 (8.3)	1
Sex at birth, *n* (%)			
Male	67 (81.7)	1070 (85.9)	.328
Origin, *n* (%)			
Spain	59 (71.1)	921(74)	.606
HIV transmission route, *n* (%)			**<**.**001**
GBMSM	50 (60.2)	838 (67.2)	
Heterosexual	13 (15.7)	259 (20.8)	
Injecting drug use	10 (12)	37 (3)	
Vertical	4 (4.8)	9 (0.7)	
Other/unknown	5 (6.1)	104 (7.3)	
CD4 nadir (cells/µL), median (IQR)	286 (161–407)	350 (207–500)	.**008**
Baseline CD4 count (cells/µL), median (IQR)	713 (562–978)	789 (586–1018)	.068
HIV-1 viral load at diagnosis (log_10_ copies/mL), median (IQR)	4.74 (4.22–5.28)	4.81 (4.24–5.33)	.766
Months from diagnosis to first ART initiation, median (IQR)	2.5 (0.6–29)	2 (0–12)	.136
Years of ART before CAB + RPV LA initiation, median (IQR)	11.5 (7.8–19)	8 (5–12)	**<**.**001**
Months of viral suppression before CAB + RPV LA initiation, median (IQR)	92 (25.5–127.8)	72 (31–120)	.290
AIDS-defining condition at baseline, *n* (%)	18 (22)	144 (11.5)	.**014**
Previous virological failure, *n* (%)	15 (18.3)	33 (2.6)	**<**.**001**
Number of blips in the 5 y prior to CAB + RPV LA initiation, *n* (%)			.081
0	61 (74.4)	903 (79.9)	
1	12 (14.6)	142 (12.6)	
2	1 (1.2)	41 (3.6)	
3	3 (3.7)	20 (1.8)	
>3	5 (6.1)	24 (2.1)	
HIV-1 subtype, *n* (%)			.763
A1/A2	1 (1.2)	21 (1.7)	
B	38 (45.8)	568 (45.5)	
F/CRF	4 (4.8)	34 (2.7)	
Other/unknown	40 (48.2)	624 (50.1)	
NRTI-RAMs, *n* (%)	37 (44.6)	10 (0.8)	**<**.**001**
M184V	16 (19.5)	8 (0.6)	<.001
Other	26 (31.7)	1 (0.1)	<.001
INSTI-RAMs, *n* (%)	1 (1.2)	0 (0)	.**004**

Abbreviations: BMI, body mass index; ART, antiretroviral therapy; CAB + RPV LA, long-acting cabotegravir plus rilpivirine; GBMSM, gay, bisexual, and other men who have sex with men; IQR, interquartile range; AIDS, acquired immunodeficiency syndrome; RAMs, resistance-associated mutations; NRTI, nucleoside/nucleotide reverse transcriptase inhibitor; NNRTI, non-nucleoside/nucleotide reverse transcriptase inhibitor; RPV, rilpivirine; INSTI, integrase strand transfer inhibitor.

Among the 97 patients with a history of NNRTI RAMS and for whom supplemental data were obtained, NNRTI RAMs were identified in baseline genotypes in 79 cases, while the remaining 18 corresponded to genotypes performed during periods of VF.

The rate of treatment discontinuation did not differ significantly between patients with non-RPV NNRTI RAMs and reference group (6.02% vs 7%; *P* = .74). The reasons for treatment discontinuation are detailed in Table 2.

**Table 2.** Clinical and Virological Outcomes During Follow-up According to NNRTI Mutations Not Affecting Rilpivirine Susceptibility

	Non-RPV NNRTI RAMs (*n* = 83)	Reference Group (*n* = 1 247)	HR (95% CI)	*P* Value
Reasons for discontinuation, *n* (%)				
Confirmed virological failure	1 (1.2)	10 (0.8)	1.39 (0.18–10.84)	.755
Local injection reaction	2 (2.4)	21 (1.7)	1.33 (0.31–5.68)	.699
Systemic adverse event	1 (1.2)	13 (1)	1.12 (0.15–8.53)	.916
Other reason	1 (1.2)	43 (3.4)	0.34 (0.05–2.51)	.293
Number of participants experiencing blips, *n* (%)	5 (6)	102 (8.2)	0.72 (0.28–1.81)	.481
Treatment adherence, *n* (%)^a^				
100%	67 (80.7)	1029 (82.5)	-	.656
90%–99.9%	15 (18.1)	196 (15.7)	-	.537
<90%	1 (1.2)	22 (1.8)	-	1
Months of follow-up, median (IQR)	16.7 (10.1–21)	14.4 (9–19.1)	-	.052
Months to discontinuation, median (IQR)^b^	0.33 (0.2–0.66)	1.55 (0–2.04)	-	.337

Abbreviations: RAMs, resistance-associated mutations; NNRTI, non-nucleoside/nucleotide reverse transcriptase inhibitor; RPV, rilpivirine; HR, hazard ratio; CI, confidence interval.

^a^Adherence to treatment was assessed as the proportion of participants who received CAB + RPV LA injections within the therapeutic window of ±7 d.

^b^Months to discontinuation: time from treatment initiation to treatment discontinuation, expressed in months.

### Virological Outcomes of Participants With Non-RPV NNRTI RAMs

**Figure 1.**
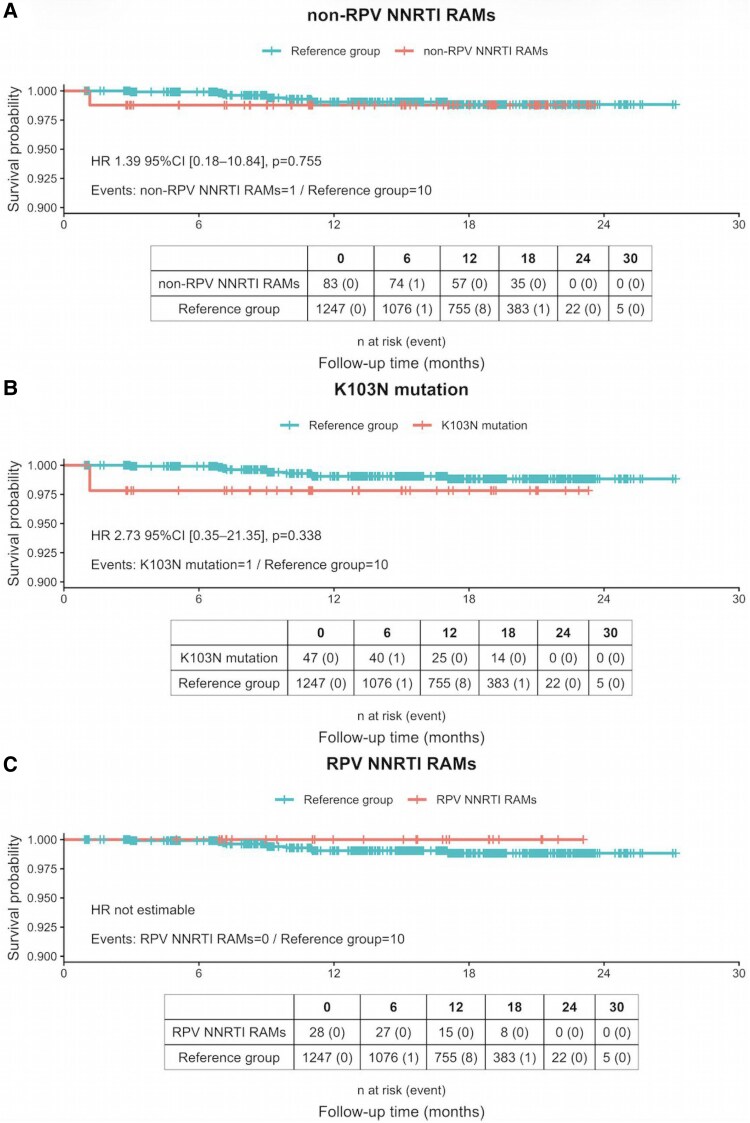
Kaplan–Meier curves for time to confirmed virological failure stratified by NNRTI mutation status. Kaplan–Meier curves showing time to confirmed virological failure according to NNRTI mutation group. *A*, Virological failures in the non-RPV NNRTI RAMs group; (*B*) virological failures in the K103N mutation group; (*C*) virological failures in the RPV NNRTI RAMs group. Hazard ratios (HRs) with 95% confidence intervals were estimated using Cox proportional hazards models, and *P* values were derived from log-rank tests. Numbers at risk are displayed below each panel. Abbreviations: RAMs, resistance-associated mutations; NNRTI, non-nucleoside/nucleotide reverse transcriptase inhibitor; RPV, rilpivirine.

After a median follow-up of 16.7 (10.1–21) months (non-RPV NNRTI RAMs) and 14.4 (9–19.1) months (reference group), we observed 11 (0.8%) CVF, 1 (1.2%) in the non-RPV NNRTI RAMs group and 10 (0.8%) in the reference population (Table 2). The rate of VF was similar between the 2 groups (1.2% vs 0.8%; HR = 1.39, 95% CI: 0.18, 10.84; *P* = .755) (Figure 1). The only VF in participants with previous non-RPV NNRTI-RAMs was observed in a patient with the K103N mutation in baseline GRT. She was a 52-year-old Spanish woman with an HIV-1 subtype B infection and a BMI of 36 kg/m^2^. She started CAB + RPV LA after 23 years of ART, including 9 years of viral suppression. Her treatment history included a prior VF with the development of M184 V and K103N mutations; no integrase strand transfer inhibitor resistance mutations were detected.

### Virological Outcomes of Participants With the K103N Mutation

There were 47 participants with the K103N mutation (3.5%). The rate of virological failure in patients with K103N versus the comparator was not significantly different (2.1% vs 0.8%; HR: 2.73, 95% CI: 0.35–21.35, *P* = .338). As previously mentioned, the only patient who had VF with NNRTI RAMs harbored the K103N mutation (Figure 1).

### Virological Outcomes of Participants With RPV NNRTI RAMs

There were 28 participants with RPV-associated NNRTI RAMs (2.1%). No virological failures were observed in participants with RAMs affecting RPV (0% vs 0.8%; *P* = 1.000) (Figure 1). Of interest, resistance to RPV in our cohort spanned the full spectrum of Stanford categories, with 17 cases of low-level or potentially low-level resistance [LLR/PLLR], 6 with intermediate-level resistance [ILR], and 5 with high-level resistance [HLR]. Table 3 summarizes the major mutations detected in the cohort and the combinations that most frequently affected non-nucleoside susceptibility. Table 4 presents the classification of these mutations according to their level of resistance and the affected antiretroviral drugs. The virological outcomes and reasons for treatment discontinuation in this subpopulation are detailed in Supplementary Table 2.

**Table 3.** Frequency of NNRTI Resistance Major Mutations and Combinations

NNRTI-RAMs	*n*	RPV-Stanford Penalty Score^a^	Resistance Interpretation
Major single mutations			
V90I	2	5	S
A98G	1	15	LLR
L100I	1	45	ILR
K103N	48	0	S
K103S	2	0	S
V106A	1	5	S
V106M	1	0	S
V108I	12	5	S
E138A	9	10	PLLR
E138K	1	45	ILR
V179D	7	10	PLLR
V179T	6	0	S
Y181C	5	45	ILR
Y181I	1	60	HLR
Y188L	1	60	HLR
G190A	1	15	LLR
G190E	1	60	HLR
H221Y	2	10	PLLR
P225H	2	0	S
F227L	3	5	S
N348I	1	0	S
Synergistic combinations			
K103N + L100I	1	60	HLR
K103N + M184V	10	0	S
K103N + V108I	3	5	S
K103N + K65R	2	0	S
K103N + Y181C	2	45	ILR
V106I + V179I	2	5	S
E138A + V179E	2	20	LLR
V106I + Y181C	1	60	HLR
K103N + P225H	1	0	S

Each mutation was counted once per patient. Combination mutations were defined as the simultaneous presence of all listed mutations within the same genotypic test. Mutations not detected in any patient included: K101P, K101H, K103H, K103T, V106L, V106M, E138G, E138Q, E138R, Y181F, Y181G, Y181S, Y188C, Y188H, G190C, G190E, G190Q, G190S, G190T, G190 V, F227Y, M230I, M230L, L234I, P236L, K238N, K238T, and Y318F.

^a^Resistance interpretation was performed using the Stanford HIV Drug Resistance Database (HIVdb), Stanford University (https://hivdb.stanford.edu).

Abbreviations: RAMs, resistance-associated mutations; NNRTI, non-nucleoside/nucleotide reverse transcriptase inhibitor; RPV, rilpivirine.

**Table 4.** NNRTI Resistance Categories According to Genotypic Resistance Scores

Drug	PLLR	LLR	ILR	HLR	Any Resistance Level
Doravirine, *n* (%)	9 (8.1)	7 (6.3)	0 (0)	5 (4.5)	21 (18.9)
Efavirenz, *n* (%)	14 (12.6)	9 (8.1)	3 (2.7)	51 (46)	77 (69.4)
Etravirine, *n* (%)	13 (11.7)	7 (6.3)	6 (5.4)	1 (0.9)	27 (24.3)
Nevirapine, *n* (%)	7 (6.3)	14 (12.6)	5 (4.5)	53 (47.8)	79 (71.2)
Rilpivirine, *n* (%)	2 (1.8)	15 (13.5)	6 (5.4)	5 (4.5)	28 (25.2)

Resistance levels were defined according to the Stanford HIVdb algorithm.

Abbreviations: PLLR, potential low-level resistance; LLR, low-level resistance; ILR, intermediate-level resistance; HLR, high-level resistance.

### Patients Experiencing Blips

**Figure 2.**
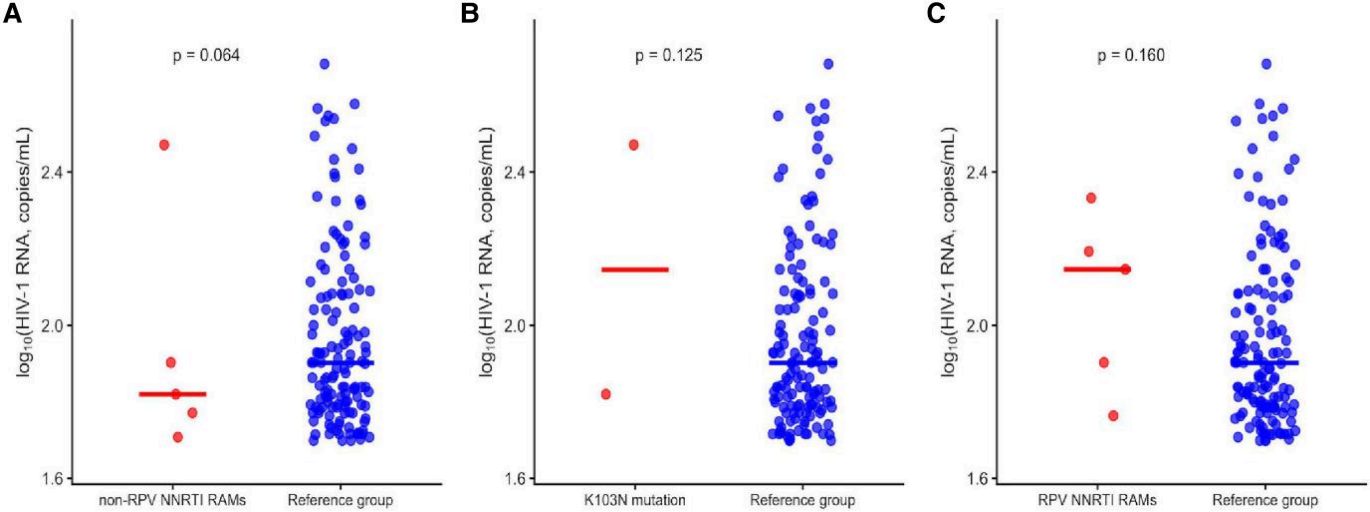
Distribution of virological blips during follow-up across study groups. The jitter plot depicts the number and dispersion of blips observed throughout follow-up among participants in each study group. *A*, non-RPV NNRTI RAMs group; (*B*) K103N mutation group; and (*C*) RPV NNRTI RAMs group. Abbreviations: RAMs, resistance-associated mutations; NNRTI, non-nucleoside/nucleotide reverse transcriptase inhibitor; RPV, rilpivirine.

During follow-up, viral blips occurred in 5 participants (6%) in the non-RPV NNRTI RAMs group, including 2 carrying the K103N mutation, and in 102 participants (8.2%) in the reference group, with no significant differences between groups (*P* = .481) (Figure 2). Baseline GRT results were available for all participants with blips except one. Among participants in the non-RPV NNRTI RAMs group, only 1 harbored acquired resistance mutations (S68G, L210W, and P225H), conferring high-level resistance to efavirenz and nevirapine. This participant experienced a single blip of 51 copies/mL at month 1 after switching to CAB + RPV LA and remained virologically suppressed thereafter. All remaining blips in this group were below 100 copies/mL, except for 1 participant with baseline K103N, V10I, F77I, and W71T mutations who presented a blip of 296 copies/mL. Of note, 4 of the 5 participants with blips in the non-RPV NNRTI RAMs group had experienced previous blips during the 5 years prior to switching to CAB + RPV LA: 2 participants had 1 previous blip, 1 participant had 2 previous blips, and 1 participant had 3 previous blips. During study follow-up, no participant experienced more than 1 blip.

Among participants with RPV-associated NNRTI RAMs, 3 experienced blips during follow-up. One participant carrying the V179D mutation presented a single blip of 80 copies/mL at month 5, without previous blips before switching to CAB + RPV LA. A second participant, with transmitted T69N, K103R, and A33F mutations and subsequently acquired K101E and E138 K mutations conferring high-level resistance to RPV, had experienced ≥3 blips during previous oral ART and presented a single blip of 156 copies/mL at month 1 after switching, followed by viral resuppression. The third participant carried the transmitted G190E mutation, also associated with high-level resistance to RPV, and had no prior history of blips. This participant experienced 3 consecutive episodes of detectable viremia (140, 215, and 58 copies/mL) before the data cutoff, although criteria for virological failure were not met (Figure 2). Adherence was evaluated in all patients with viral blips. All patients except one received treatment within the scheduled dosing window. The only patient with suboptimal adherence had delays in 2 injections, although their proportion of days covered remained >90%, indicating a high overall level of treatment adherence.

## DISCUSSION

In this real-world study, CAB + RPV LA demonstrated similar rates of virologic suppression among PWH with a previous history of non-RPV NNRTI RAMs detected in plasma HIV-RNA compared with those without prior NNRTI RAMs.

Consistent with our findings, other reports and studies evaluating oral dual combinations of RPV with integrase inhibitors have described good effectiveness in people with non-RPV NNRTI RAMs [8, 9, 23–26]. To our knowledge, this is the largest real-world cohort published to date assessing the effectiveness of CAB + RPV LA in patients harboring NNRTI RAMs. Other real-world studies have reported heterogeneous results regarding both the number of patients with NNRTI-associated mutations receiving this regimen and the characterization of these mutations [8, 9, 13, 17, 24]. Moreover, detailed characterization of these mutations, including resistance levels, drug-specific effects, and differentiation from polymorphisms, remains scarce [9, 13, 14, 26]. In the CARES study [23], conducted in low- and middle-income countries, sustained virologic suppression with CAB + RPV LA remained exceptionally high (97%) despite the detection of archival NNRTI resistance mutations, including RPV-associated mutations, by next-generation sequencing of proviral DNA from peripheral blood mononuclear cells. These findings add to growing evidence that archival resistance detected in the viral reservoir does not necessarily predict VF in clinically stable, virologically suppressed individuals switching therapy [27, 28]. In such patients, durable treatment success is frequently maintained despite the presence of low-frequency or historical resistance mutations affecting components of the regimen. Importantly, our findings extend this evidence by demonstrating high effectiveness based on documented historical genotypes rather than proviral DNA analyses [27, 28]. This distinction is clinically relevant because current European labeling for CAB + RPV LA contraindicates its use in individuals with any prior NNRTI RAMs, irrespective of its predicted impact on RPV susceptibility [18].

We also observed no differences in the rate of blips between the groups (6% vs 8.2%; *P* = .481). These findings are consistent with real-world evidence reported by Gutiérrez et al., who described the high effectiveness of CAB + RPV LA in patients with RPV NNRTI RAMs and found no increase in viral blips or intermittent episodes of detectable viremia [13, 29]. These data reinforce the notion that CAB + RPV LA maintains similar potency regardless of the presence of NNRTI RAMs.

The rate of VF in our study was very low in both groups (0.8% vs 1.2%) and comparable to that reported in pivotal clinical trials [2–5, 17, 23]. Cutrell et al reported in a pooled post hoc analysis of 1651 participants from the phase 3/3b FLAIR, ATLAS, and ATLAS-2 M studies that CVF was rare (1.4% through up to 152 weeks) [16]. Confirming these findings in real-world clinical practice is important because outcomes observed under controlled trial conditions may differ from those observed in routine care.

We also evaluated the clinical relevance of the K103N mutation, one of the most frequently selected NNRTI RAMs in individuals who experienced VF with regimens containing nevirapine or efavirenz. Given the widespread historical use of these drugs, K103N remains one of the most prevalent NNRTI RAMs in both treatment-naïve (as transmitted resistance) and treatment-experienced PWH [30]. Our data demonstrate similar effectiveness among PWH harboring this mutation. This information may have meaningful clinical implications especially in Europe, potentially expanding the number of patients eligible for this regimen.

In our series, VF occurred in only 1 patient with non-RPV NNRTI RAMs. Specifically, this case involved a 52-year-old woman with a body mass index of 36 kg/m^2^ and a 23-year history of ART. She had a history of VF with documented K103N and M184 V mutations. This case likely represents a complex clinical scenario characterized by extensive treatment exposure and prior failures, suggesting that overall clinical complexity may be more relevant than the isolated presence of the K103N mutation.

Finally, we analyzed 28 patients with RPV NNRTI RAMs, including 5 participants with predicted high-level RPV resistance. After a median follow-up of 13.5 (7.3–18.9) months, no VF was observed. Notably, the previously described participant with HLR presented 3 detectable viral loads before the data cutoff, although criteria for virological failure were not met at that time. After completion of the present analysis, this participant was later reported to have met criteria for virological failure and to have developed additional mutations associated with CAB resistance. These individuals are not typically considered candidates for this regimen. However, as this was an observational real-world study, we were unable to determine the clinical rationale for treatment selection. Therefore, these findings should be interpreted cautiously, given the small sample size and limited follow-up. While initiating CAB + RPV LA in patients with NNRTI mutations that preserve RPV susceptibility may be clinically reasonable, its use in individuals with established RPV RAMs cannot be recommended based on the current evidence [31–33]. Multivariable analyses have consistently identified RPV RAMs as major risk factors for CAB + RPV LA failure [15, 16].

Our study had several limitations. First, key variables, including prior VF and RAMs, were collected retrospectively, introducing the potential for information bias. Second, the relatively small number of participants with NNRTI RAMs reflects real-world prescribing practices but limits the subgroup analyses. Given the high proportion of participants with baseline RAMs and the limited number of patients with mutations emerging after VF, these findings cannot be generalized to patients with prior VF. Third, follow-up procedures were not standardized across the centers, resulting in heterogeneity in data collection. Finally, the observational design precludes causal inference, and residual confounding cannot be excluded despite the predefined analytical criteria.

This study also has several strengths. First, this nationwide multicenter study included 58 sites across Spain, capturing substantial heterogeneity in clinical practice and enhancing its external validity. Second, the reference group was carefully defined to minimize confounding. Participants with characteristics associated with an increased risk of VF, including detectable viremia, subtype A6/A1, and prior VF, were excluded [15, 16]. Third, this interim analysis includes a detailed characterization of RAMs and their predicted impact on drug susceptibility. Finally, CVF was defined as a single HIV-1 RNA measurement ≥500 copies/mL, a more stringent criterion than that used in the current consensus guidelines [22].

The favorable outcomes observed are particularly relevant given that most NNRTI RAMs identified were transmitted rather than acquired during the prior treatment failure. Consistent with this concern, WHO reports have highlighted the increasing global prevalence of transmitted NNRTI resistance, including mutations affecting RPV [30, 34].

In conclusion, CAB + RPV LA demonstrated high and durable virologic effectiveness in carefully selected PWH harboring NNRTI RAMs that were not predicted to compromise RPV activity. In the absence of major baseline risk factors for VF, including clinically relevant RPV resistance, HIV-1 subtype A6/A1, or multiple concurrent risk determinants, the presence of certain NNRTI mutations did not adversely affect treatment outcomes. Nevertheless, given the observational design, limited number of resistance cases, and global prevalence of transmitted NNRTI resistance, these findings should be interpreted with caution. Additional data from larger and more heterogeneous cohorts are required.

## Supplementary Material

ofag426_Supplementary_Data

## Supplementary Data

Supplementary materials are available at *Open Forum Infectious Diseases* online. Consisting of data provided by the authors to benefit the reader, the posted materials are not copyedited and are the sole responsibility of the authors, so questions or comments should be addressed to the corresponding author.

## References

[1] Swindells S, Lutz T, Van Zyl L, et al Week 96 extension results of a phase 3 study evaluating long-acting cabotegravir with rilpivirine for HIV-1 treatment. AIDS Lond Engl 2022; 36:185–94. 10.1097/QAD.0000000000003025 PMC8711605 34261093

[2] Jaeger H, Overton ET, Richmond G, et al Long-acting cabotegravir and rilpivirine dosed every 2 months in adults with HIV-1 infection (ATLAS-2M), 96-week results: a randomised, multicentre, open-label, phase 3b, non-inferiority study. Lancet HIV 2021; 8:e679–89. 34648734 10.1016/S2352-3018(21)00185-5

[3] Overton ET, Richmond G, Rizzardini G, et al Long-acting cabotegravir and rilpivirine dosed every 2 months in adults with human immunodeficiency virus 1 type 1 infection: 152-week results from ATLAS-2M, a randomized, open-label, phase 3b, noninferiority study. Clin Infect Dis 2023; 76:1646–54. 36660819 10.1093/cid/ciad020 PMC10156123

[4] Swindells S, Andrade-Villanueva JF, Richmond GJ, et al Long-acting cabotegravir and rilpivirine for maintenance of HIV-1 suppression. N Engl J Med 2020; 382:1112–23. 32130809 10.1056/NEJMoa1904398

[5] Orkin C, Arasteh K, Górgolas Hernández-Mora M, et al Long-acting cabotegravir and rilpivirine after oral induction for HIV-1 infection. N Engl J Med 2020; 382:1124–35. 32130806 10.1056/NEJMoa1909512

[6] Overton ET, Richmond G, Rizzardini G, et al Long-acting cabotegravir and rilpivirine dosed every 2 months in adults with HIV-1 infection (ATLAS-2M), 48-week results: a randomised, multicentre, open-label, phase 3b, non-inferiority study. Lancet Lond Engl 2021; 396:1994–2005. 10.1016/S0140-6736(20)32666-0 33308425

[7] Orkin C, Oka S, Philibert P, et al Long-acting cabotegravir plus rilpivirine for treatment in adults with HIV-1 infection: 96-week results of the randomised, open-label, phase 3 FLAIR study. Lancet HIV 2021; 8:e185–96. 33794181 10.1016/S2352-3018(20)30340-4

[8] Gagliardini R, De Benedittis S, Tavelli A, et al Effectiveness and predictors of treatment discontinuation of long-acting cabotegravir/rilpivirine in virologically suppressed people with HIV: real-life data from the icona cohort. J Antimicrob Chemother 2025; 80:2169–78. 40570134 10.1093/jac/dkaf184 PMC12313467

[9] Sension MG, Brunet L, Hsu RK, et al Cabotegravir + rilpivirine long-acting injections for HIV treatment in the US: real world data from the OPERA cohort. Infect Dis Ther 2023; 12:2807–17. 37966701 10.1007/s40121-023-00890-2 PMC10746614

[10] Lowenthal ED, Chapman J, Ohrenschall R, et al Acceptability and tolerability of long-acting injectable cabotegravir or rilpivirine in the first cohort of virologically suppressed adolescents living with HIV: results from the more options for children and adolescents (IMPAACT 2017/MOCHA) phase I/II trial. Lancet HIV 2024; 11:e222–32. 38538161 10.1016/S2352-3018(23)00301-6 PMC11061207

[11] Buzon MJ, Martin-Gayo E, Pereyra F, et al Long-term antiretroviral treatment initiated at primary HIV-1 infection affects the size, composition, and decay kinetics of the reservoir of HIV-1-infected CD4 T cells. J Virol 2014; 88:10056–65. 24965451 10.1128/JVI.01046-14 PMC4136362

[12] Fernández-González M, Telenti G, Ledesma C, et al Influence of patient characteristics and oral lead-in on long-acting cabotegravir and rilpivirine pharmacokinetics and outcomes in people with HIV: a real-world study. Antimicrob Agents Chemother 2025; 69:e00145–25. 40470947 10.1128/aac.00145-25 PMC12217485

[13] Gutiérrez F, Fernández-González M, Ledesma C, et al Virological history predicts non-sustained viral suppression with long-acting cabotegravir and rilpivirine therapy, independent of pharmacokinetic parameters. Clin Infect Dis 2025; 80:842–53. 39298641 10.1093/cid/ciae475

[14] van Welzen BJ, Van Lelyveld SFL, Ter Beest G, et al Virological failure after switch to long-acting cabotegravir and rilpivirine injectable therapy: an in-depth analysis. Clin Infect Dis 2024; 79:189–95. 38207125 10.1093/cid/ciae016 PMC11259215

[15] Orkin C, Schapiro JM, Perno CF, et al Expanded multivariable models to assist patient selection for long-acting cabotegravir + rilpivirine treatment: clinical utility of a combination of patient, drug concentration, and viral factors associated with virologic failure. Clin Infect Dis 2023; 77:1423–31. 37340869 10.1093/cid/ciad370 PMC10654860

[16] Cutrell AG, Schapiro JM, Perno CF, et al Exploring predictors of HIV-1 virologic failure to long-acting cabotegravir and rilpivirine: a multivariable analysis. AIDS Lond Engl 2021; 35:1333–42. 10.1097/QAD.0000000000002883 PMC8270504 33730748

[17] Jongen VW, Wit FWNM, Boyd A, et al Effectiveness of bi-monthly long-acting injectable cabotegravir and rilpivirine as maintenance treatment for HIV-1 in The Netherlands: results from the Dutch ATHENA national observational cohort. Lancet HIV 2025; 12:e40–50. 39779044 10.1016/S2352-3018(24)00269-8

[18] Rekambys medical label. Available at: https://www.ema.europa.eu/en/documents/product-information/rekambys-epar-product-information_en.pdf. Accessed June 1, 2026.

[19] Buzón-Martín L, Montes ML, Pedrero R, et al A prospective assessment of the efficacy and durability of long-acting cabotegravir and rilpivirine in individuals with HIV in Spain (RELATIVITY study). J Antimicrob Chemother 2025; 23:dkaf389. 10.1093/jac/dkaf389 41128149

[20] Wensing AM, Calvez V, Ceccherini-Silberstein F, et al 2025 update of the drug resistance mutations in HIV-1. Top Antivir Med **2025**; 33:457-473. PMID: 40472382. 40472382

[21] HIV Drug Resistance Database. Stanford University. Available at: https://hivdb.stanford.edu. Accessed June 1, 2026.

[22] Orkin C, Paterson A, Elias A, et al Establishing shared definitions of virological failure and discontinuation for long-acting injectable cabotegravir and rilpivirine therapy (the CONSENSUS-LAI study): an international survey and Delphi process. Lancet HIV 2025; 12:e649–59. 40774264 10.1016/S2352-3018(25)00131-6

[23] Kityo C, Mambule IK, Musaazi J, et al Switch to long-acting cabotegravir and rilpivirine in virologically suppressed adults with HIV in Africa (CARES): week 48 results from a randomised, multicentre, open-label, non-inferiority trial. Lancet Infect Dis 2024; 24:1083–92. 38821073 10.1016/S1473-3099(24)00289-5

[24] Serris A, Ferre VM, Le Hingrat Q, et al Real-world data on long-acting intramuscular maintenance therapy with cabotegravir and rilpivirine mirror phase 3 results. J Antimicrob Chemother 2024; 79:2932–8. 39287977 10.1093/jac/dkae308

[25] Ramgopal MN, Castagna A, Cazanave C, et al Efficacy, safety, and tolerability of switching to long-acting cabotegravir plus rilpivirine versus continuing fixed-dose bictegravir, emtricitabine, and tenofovir alafenamide in virologically suppressed adults with HIV, 12-month results (SOLAR): a randomised, open-label, phase 3b, non-inferiority trial. Lancet HIV 2023; 10:e566–77. 37567205 10.1016/S2352-3018(23)00136-4

[26] Moyle G, Assoumou L, De Castro N, et al Durable efficacy of dolutegravir/rilpivirine switch in subjects with HIV RNA <50 copies/ml and history of K103N mutation. AIDS. 2026. AIDS 2022; 40:885–93. 10.1097/QAD.0000000000004451 41587441

[27] Andreatta K, Willkom M, Martin R, et al Switching to bictegravir/emtricitabine/tenofovir alafenamide maintained HIV-1 RNA suppression in participants with archived antiretroviral resistance including M184V/I. J Antimicrob Chemother 2019; 74:3555–64. 31430369 10.1093/jac/dkz347 PMC6857193

[28] De Miguel R, Rial-Crestelo D, Dominguez-Dominguez L, et al Dolutegravir plus lamivudine for maintenance of HIV viral suppression in adults with and without historical resistance to lamivudine: 48-week results of a non-randomized, pilot clinical trial (ART-PRO). EBioMedicine 2020; 55:102779. 32408111 10.1016/j.ebiom.2020.102779 PMC7225620

[29] Gutiérrez F, Fernández-González M, Ledesma C, et al Virological success with long-acting cabotegravir and rilpivirine despite historical rilpivirine resistance: a real-world case series. Open Forum Infect Dis 2025; 12:ofaf666. 41234301 10.1093/ofid/ofaf666 PMC12611297

[30] World Health Organization . HIV drug resistance report, 2024. Available at: https://iris.who.int/server/api/core/bitstreams/b9c5dba5-4055-46e3-ab42-abdd00f94aa5/content. Accessed December 12, 2025.

[31] GeSIDA Spanish HIV Guidelines. Available at: https://guiasclinicas.gesida-seimc.org/version/?do=show&pk=24. Accessed December 13, 2025.

[32] European AIDS Clinical Society HIV Guidelines v.13. Available at: https://eacs.sanfordguide.com. Accessed December 13, 2025.

[33] Gandhi RT, Landovitz RJ, Sax PE, et al Antiretroviral drugs for treatment and prevention of HIV in adults: 2024 recommendations of the international antiviral society–USA panel. JAMA 2025; 333:609–28. 39616604 10.1001/jama.2024.24543

[34] Bertagnolio S, Hermans L, Jordan MR, et al Clinical impact of pretreatment human immunodeficiency virus drug resistance in people initiating nonnucleoside reverse transcriptase inhibitor–containing antiretroviral therapy: a systematic review and meta-analysis. J Infect Dis 2020; 224:377–88. 10.1093/infdis/jiaa683 PMC8328216 33202025

