# Supplementary material for: Effect of Non-nucleoside Resistance-Associated Mutations on the Effectiveness of Long-Acting Cabotegravir + Rilpivirine Therapy: Insights From the Real-World RELATIVITY Cohort
Source: Open Forum Infect Dis. 2026 Jul 7;13(7):ofag426. doi: 10.1093/ofid/ofag426 (PMC13371753; doi:10.1093/ofid/ofag426)
Supplement: ofag426_Supplementary_Data [file ofag426_supplementary_data.docx]

**Supplementary Table 1.** **Baseline Characteristics According to RPV resistance-associated mutations.**

|  | **RPV NNRTI RAMs**  (n = 28) | **Reference Group**  (n = 1 247) | ***P***  **value** |
| --- | --- | --- | --- |
| **Age (years),** median (IQR) | 41.5 (33-53.2) | 44.0 (37.0-52.0) | .346 |
| **BMI (Kg/m^2^),** median (IQR) | 23.9 (20.7-26.1) | 25.0 (22.4-27.8) | .062 |
| **BMI ≥ 30 Kg/m^2^,** n (%) | 0 (0) | 167 (14.6) | .038 |
| **Oral lead-in therapy**, n (%) | 1 (3.6) | 103/1247 (8.3%) | .722 |
| **Sex at birth,** n (%) |  |  |  |
| Male | 24 (85.7) | 1070 (85.9) | 1 |
| **Origin**, n (%) |  |  | . |
| Spain | 20 (71.4) | 921 (74) | .828 |
| **HIV transmission route**, n (%) |  |  | .482 |
| GBMSM | 16 (57.1) | 838 (67.2) |  |
| Heterosexual | 5 (17.9) | 259 (20.8) |  |
| Injecting drug use | 2 (7.1) | 37 (3) |  |
| Vertical | 1 (3.6) | 9 (0.7) |  |
| Other/Unknown | 4 (14.3) | 29 (2.3) |  |
| **CD4 nadir (cells/µL)**, median (IQR) | 444 (187-551) | 350 (207-500) | .477 |
| **Baseline CD4 count (cells/µL)**, median (IQR) | 748 (573 -932) | 789 (586 -1018) | .496 |
| **HIV-1 viral load at diagnosis (log_10_ copies/mL),** median (IQR) | 4.98 (4.1–5.4) | 4.81 (4.24–5.33) | .553 |
| **Months from diagnosis to first ART initiation**, median (IQR) | 2 (0.1-8) | 2 (0-12) | .751 |
| **Years of ART before CAB+RPV LA initiation**, median (IQR) | 8 (6.2-11) | 8 (5-12) | .944 |
| **Months of viral suppression before CAB+RPV LA initiation**, median (IQR) | 84 (44-112) | 72 (31-120) | .734 |
| **AIDS-defining condition at baseline**, n (%) | 2 (7.1) | 144 (11.5) | .762 |
| **Previous virological failure**, n (%) | 4 (14.3) | 33 (2.6) | **.007** |
| **Number of blips in the 5 years prior to CAB+RPV LA initiation**, n (%) |  |  | .287 |
| 0 | 24 (92.3) | 903 (79.9) |  |
| 1 | 1 (3.8) | 142 (12.6) |  |
| 2 | 0 (0) | 41 (3.6) |  |
| 3 | 0 (0) | 20 (1.8) |  |
| >3 | 1 (3.8) | 24 (2.1) |  |
| **HIV-1 subtype**, n (%) |  |  | **.031** |
| A1/A2 | 3 (10.7) | 21 (1.7) |  |
| B | 12 (42.9) | 568 (45.5) |  |
| F/CRF | 2 (7.1) | 34 (2.7) |  |
| Other/Unknown | 11 (39.3) | 624 (50.1) |  |
| **NRTI-RAMs**, n (%) | 5 (17.9) | 10 (0.8) | **<.001** |
| M184V | 1 (3.6) | 8 (0.6) | .182 |
| Other | 6 (21.4) | 1 (0.1) | **<.001** |
| **INSTI-RAMs**, n (%) | 0 (0) | 0 (0) |  |

*Abbreviations: BMI, body mass index; ART, antiretroviral therapy; CAB+RPV LA, long-acting cabotegravir plus rilpivirine; GBMSM, gay, bisexual, and other men who have sex with men; IQR, interquartile range; AIDS, acquired immunodeficiency syndrome; RAMs, resistance-associated mutations; NRTI, nucleoside/nucleotide reverse transcriptase inhibitor; NNRTI, non-nucleoside/nucleotide reverse transcriptase inhibitor; RPV, rilpivirine; INSTI, integrase strand transfer inhibitor.*

**Supplementary Table 2.** **Clinical and Virological Outcomes During Follow-up According to RPV resistance-associated mutations.**

|  | **RPV NNRTI RAMs**  (n = 28) | **Reference Group**  (n = 1 247) | **HR** (95% CI) | ***P***  **value** |
| --- | --- | --- | --- | --- |
| **Reasons for discontinuation,** n (%) |  |  |  |  |
| Confirmed virological failure | 0 (0) | 10 (0.8) | - | - |
| Local injection reaction | 0 (0) | 21 (1.7) | - | - |
| Systemic adverse event | 1 (3.6) | 13 (1) | 3.23 (0.42-24.66) | .259 |
| Other reason | 0 (0) | 43 (3.4) | - | - |
| **Number of participants experiencing blips,** n (%) | 3 (10.7) | 102 (8.2) | 1.33 (0.52–3.44) | .552 |
| **Treatment adherence,** n (%)^a^ |  |  |  |  |
| 100% | 22 (78.6) | 1029 (82.5) | - | .614 |
| 90–99.9% | 5 (17.9) | 196 (15.7) | - | .792 |
| <90% | 1 (3.6) | 22 (1.8) | - | .403 |
| **Months of follow-up,** median (IQR) | 13.5 (7.3-18.9) | 14.4 (9.0-19.1) | - | .837 |
| **Months to discontinuation,** median (IQR) ^b^ | 0 (0-0) | 1.55 (0-2) | - | .249 |

*Abbreviations: RAMs, resistance-associated mutations; NNRTI, non-nucleoside/nucleotide reverse transcriptase inhibitor; RPV, rilpivirine; HR, hazard ratio; CI, confidence interval.*

*^a^* *Adherence to treatment was assessed as the proportion of participants who received CAB+RPV LA injections within the therapeutic window of ±7 days.*

^b^ *Months to discontinuation: time from treatment initiation to treatment discontinuation, expressed in months.*
